# Autoantibodies to tumor-associated antigens as biomarkers in human hepatocellular carcinoma (HCC)

**DOI:** 10.1186/2162-3619-2-15

**Published:** 2013-05-20

**Authors:** Liping Dai, Ningjing Lei, Mei Liu, Jian-Ying Zhang

**Affiliations:** 1 Department of Biological Sciences, The University of Texas at El Paso, El Paso, TX 79968, USA

**Keywords:** Hepatocellular carcinoma, Tumor associate antigen, Autoimmunity, Biomarker, Immunoserological diagnosis

## Abstract

Tumor-associated antigens (TAAs) recognized by cellular and/or humoral effectors of the immune system are attractive targets for diagnostic and therapeutic approaches to human cancer. Different approaches can be used to comprehensively characterize and validate the identified TAA/anti-TAA systems, which are potential biomarkers in cancer immunodiagnosis. Hepatocellular carcinoma (HCC) is one of the most common cancers worldwide. The high fatality rate of HCC within one year after its detection might be partly attributed to a lack of diagnostic methods that enable the early detection. Our previous studies have shown that novel autoantibodies can appear which are not detected prior to pre-malignant conditions during transition from chronic liver disease to HCC. The hypothesis we advance is the transition to malignancy can be associated with autoantibody response to certain cellular proteins that might have some role in tumorigenesis. We propose that the information that the cancer patient’s immune system is conveying in the form of autoantibodies to tumor-associated antigens (TAAs) should be utilized to a greater extent in identifying early signs of tumorigenesis. In this review, we will focus on the important features of TAA and the possibility that autoantibodies to TAAs can be used as biomarkers in immunodiagnosis and prognosis of HCC.

## Introduction

Hepatocellular carcinoma (HCC) is the third most common cause of cancer-related death worldwide. The majority of people with HCC will die within one year since they have been made a definite diagnosis. The high fatality rate of HCC can partly be attributed to a lack of sensitive detection method for HCC early diagnosis. Serum alpha fetoprotein (AFP) is the most effective marker available to detect HCC, however the sensitivity and specificity are not optimal, especially in patients with small tumors or in well-to-moderately differentiated HCC [1-3]. Therefore, it is necessary to develop more sensitive and specific methods for supplementing AFP in the early detection of HCC.

There are many studies demonstrating that the immune system can recognize the antigenic changes in cancer cells, and further develop autoantibodies against these antigens which have been called tumor-associated antigens (TAAs) [4,5]. Therefore, these cancer-associated autoantibodies might be considered as “reporters” from the immune system, to identify the antigenic changes of cellular proteins involved in the transformation process [2,5]. Research on autoantibody immunity to cancer-associated proteins has received great attention. As the detection of antibody immunity to tumor antigens becomes more routine, investigators have begun to address specific clinical application of cancer-associated autoantibody as a marker for detection of cancer, as a tool to monitor therapy, or as an indicator to predict disease prognosis. With the expending list of autoantibodies to TAAs, the identification of cancer-associated autoantibody signatures may therefore become useful as a tool for cancer diagnosis and prognosis [6-8].

The purpose of this review will focus on the recent advances in our studies primarily associated with the idea and possibility that detection of anti-TAAs autoantibodies can be useful for HCC immunodiagnosis.

This study was approved by the Institutional Review Board of The University of Texas at El Paso and collaborating institutions.

## Tumor-associated antigens (TAAs) and autoantibodies

One of the most extensively studied TAAs is p53, the tumor suppressor protein, which is a phosphoprotein barely detectable in the nucleus of normal cells [9]. P53 can arrest cell-cycle progression on cellular stress, particularly induced by DNA damage, to allow the DNA to be repaired [10] or trigger a process that can lead to cell apoptosis [11]. Autoantibodies to p53 in cancer were first reported in 1982 [12] and since then there have been numerous reports confirming and extending this finding [13]. Antibodies to p53 have now been found in many types of cancer, including lung [14], esophageal [15], oral [16], colon [17], gastric [18], hepatic [19], breast [20] cancers. A review article demonstrated that p53 antibodies are a specific marker of patients with malignancy [13]. For several tumor sites a decline or even disappearance of p53 antibody levels few weeks after surgical tumor removal has been demonstrated, which is in line with the hypothesis that constant stimulation of the immune system by the antigen is necessary to maintain high antibody levels [21-24]. These findings led to the suggestion that antibody serology might be a tool for detection of disease recurrence [25].

The types of cellular proteins inducing autoantibody responses are quite varied and include oncogene products such as HER-2/neu [26], cellular proteins which shield mRNAs from natural physiological degradation such as p62/IMP2 [27] and CRD-BP [28], onconeural antigens in the paraneoplastic disorder syndrome [29], differentiation-antigens such as tyrosinase and cancer/tetis antigens [30].

The approach used in our laboratory to identify TAAs has involved initially examining the sera from cancer patients using extracts of tissue culture cells as source of antigens in Western blotting or by indirect immunofluorescence (IIF) on whole cells. With these two techniques, sera with high-titered fluorescent staining and strong signals to cell extract have been identified and subsequently used as probes to immunoscreen cDNA expression libraries and isolate cDNA clones encoding targeted antigens. HCC1 [31], p62/IMP2 [27], p90 [32] and other several novel TAAs have been identified in this manner.

A major issue in the field of cancer immunodiagnosis is the definition of what constitutes a TAA. It is erroneous to include all cellular antigens identified by autoantibodies in cancer sera as TAAs, since some autoantibodies may exist in conditions that pre-date malignancy. Many antibodies to autologous cellular proteins can be detected in the sera of patients with cancer, and the reactive antigens have been called TAAs without considering the possibility that the autoantibodies might have existed in the patient before cancer. It indicates that autoantibodies from a cancer patient obtained at only one time point would not be necessarily represent immune responses to a TAA. Failing to recognize the likelihood of pre-malignant circulating antibodies would result in the inclusion of many antigens erroneously as TAAs. Further research should be needed to establish true tumor-related antigens and to exclude those that might have been present prior to malignancy. In the further analysis, anti-TAA autoantibodies may become useful diagnostic biomarkers only after it has undergone extensive trials with many cancer sera and also with many non-cancer control sera [33].

Hepatocellular carcinoma (HCC) is one of unique cancers since it could be followed up from a cohort of patients with chronic liver disease who will likely develop malignancy over a period time more than one decade. During transition from chronic liver disease to malignancy, novel autoantibodies have been observed to appear which are not detected prior to pre-malignant conditions [34]. As mentioned above, our previous studies have identified several novel TAAs including HCC1 [31], p62/IMP2 [27] and p90 [32], which might be potential biomarkers in cancer immunodiagnosis. Below will be a brief discussion of the identification and significance of each TAA we have identified in HCC.

### HCC1/CAPER

HCC1/CAPER is the first HCC-associated TAA isolated and identified [31]. Serum from a patient NK at the time when cancer was diagnosed was used to immunoscreen a HepG2 cell line λ - zap cDNA expression library in attempt to clone genes encoding any of the protein antigens. HCC1/CAPER, one of six cDNA clones which were all different-length isolates, has a full-length sequence, encoding a protein of 530 amino acid and migrating at 64 kDa in SDS-PAGE. HCC1/CAPER was a novel gene with unknown function at that time since the nucleotide and protein sequences were not found in Genbank and EMBL and SwissProt databanks. HCC1/CAPER contained a RS (arginine-serine) motif in the NH-terminus and three RNA-binding, RNP-CS (ribonucleoprotein consensus sequence, which has paired RNP1 and RNP2 regions) in the rest of the molecule. The structural peptide motifs of HCC1/CAPER were most similar to U2AF65, a smaller protein of 475 amino acid whose function was already characterized and shown to be an alternative splicing factor involved in pre-mRNA processing [31]. Other proteins with somewhat similar domain structure, such as SC35 [35] and SF2/ASF [36] and SRp55 [37], were also splicing factors involved in mRNA processing. However, the role of HCC1/CAPER in constitutive splicing is poorly understood. And there was no indication as to how it might be involved in tumorigenesis.

Nine years later, Jung et al reported that HCC1/CAPER was a co-activator of activating protein-1 and estrogen receptors and proposed naming it CAPER [38]. Three years after that, O’Malley and his colleagues shows that HCC1/CAPER was also an alternative splicing factor involved in the regulation of *calcitonin* and Vascular endothelial growth factor (*VEGF)* gene expression [39,40], confirming function that was expected of its peptide motifs. More interestingly, a recent study has reported that HCC1/CAPER may an important role in modulating the oncogenic activity of Rel/NF-kB, suggesting that a dominant-negative mutant of HCC1/CAPER enhanced vRel-mediated transformation [41]. In a recent study, autoantibody responses to HCC1 were extensively evaluated in sera from patients with HCC, liver cirrhosis and chronic hepatitis, and other type of cancer. The prevalence of autoantibodies against HCC1 was 20.1% in HCC sera, which was significantly higher than that in NHS (2.3%), but not for other type of cancer (unpublished data). With the further understanding of the function of HCC1/CAPER, we may consider HCC1/CAPER as a candidate TAA. Actually, it is almost 15 years later after the first report of the identification of HCC1/CAPER in liver cancer.

### p62/IMP2 (IGF-II mRNA binding protein)

p62/IMP2 was firstly identified as an autoantigen by screening the sera from patients with HCC [27]. Later studies found that p62/IMP2 is a member of the IGF-II mRNA binding protein (IMP) family, containing four hnRNP K-homology (KH) domains and two RNA recognition motifs [42]. It is encoded by a splice variant of the IMP2 gene, thus is known as an IMP2 isoform. The IMP family (IMP1, IMP2 and IMP3) binds and regulates translation of insulin-like growth factor 2 mRNA. Members of this family are oncofetal proteins [43], which have been implicated in RNA localization, stability and translation that are essential for normal embryonic growth and development. The expression disappears from all tissues soon after birth but frequently re-express during the process of malignant transformation. The overexpression of these proteins has been detected in many types of tumors, [27,44,45] and it has been hypothesized that the proteins can mediate cell motility and invasion, and might be closely related to cancer.

Antibody to p62/IMP2 was present in 21% of patients with HCC but not in the precursor conditions of chronic hepatitis and liver cirrhosis [27]. Other proteins from the IMP family also show certain relationship to malignancy. IMP1, which is almost identical to mouse coding region determinant-binding protein (CRD-BP) [46] and closely related to chicken zip-code binding protein 1 (ZBP1), binds directly to and stabilizes oncogenic c-Myc and regulates its posttranscriptional expression and translation [47,48]. IMP3 is orthologous to Xenopus Vg1RBP/Vera and mouse K-homology protein overexpressed in cancer (KOC), which has been shown to be transcriptionally overexpressed in cancer [49].

### p90/CIP2A (Cancerous Inhibitor of PP2A)

CIP2A is another tumor-associated antigen migrating in SDS-PAGE gel as a protein of 90 kD (also known as p90) [32]. It was firstly identified when screening sera from patients with gastric and liver cancer [32]. This protein has been shown to inhibit PP2A (Protein phosphatase 2) function, as a result stabilize the oncogene c-myc in human malignancies*.* Phosphorylation is a key kind of posttranscriptional modifications, which is also critical for the process of malignancy. PP2A exerts its tumor suppressive effects through dephosphorylating substrates at Serine and Threonine residues. Current understandings of the role of p90/CIP2A in tumor formation and progression mainly focus on its ability to inhibit PP2A phosphatase activity to regulate c-myc stability [50]. Several groups have confirmed the importance of p90/CIP2A in tumor formation in different cancer types like breast cancer, prostate cancer, gastric cancer, non-small cell lung cancer, oral carcinoma, esophageal squamous cell carcinoma and chronic myeloid leukemia [51-57].

## Approaches to TAAs identification

Autoantibodies to TAAs have been reported in a variety of human cancers, providing an insight into the interplay between malignancies and the immune response, and giving rise to novel diagnostic and therapeutic concepts [5]. Experimental technologies for identification of tumor-associated antigens include display methods such as phage display, serological expression cloning analysis, and TAA arrays [58,59]. The approaches we once used in identifying the TAAs involved examining the sera of cancer patients using extracts of tissue or cultured cells as a source of antigens in Western blotting or by indirect immunofluorescence on whole cells. These two commonly used techniques have helped us to identify sera which have strong signals on Western blotting or high fluorescent staining, and subsequently use the antibodies in these sera to isolate cDNA clones from cDNA expression libraries. In this case, several novel TAAs including HCC1/CAPER [31], SG2NA [60], p62/IMP2 [27], p90/CIP2A [32] were identified.

Serological analysis of recombination cDNA expression libraries (SEREX) [61] is a useful methodology in identifying tumor antigens to autoantibodies from patients with different types of cancer [5], which is a modified technology of our previously used approach [27,31,32,60]. The rational of this method is that intracellular proteins which are involved in carcinogenesis are provoking autoantibody responses and therefore autoantibodies can be used to immunoscreen cDNA expression libraries to isolate, identify and characterize proteins which might potentially be involved in malignant transformation. SEREX has proven to be a powerful tool in identifying antibody targets in a number of tumor types, including lymphoma, leukemia, esophageal cancer, gastric cancer, etc. [62,63]. The number of SEREX-defined antigens exceeds 2000 kinds, with approximately one-third defining novel genes at the time of their discovery [64]. Our approach was not restricted to use of cDNA libraries from autologous tumors because our previous studies had shown that antibodies in HCC sera were reactive with antigens expressed by a variety of tissue culture cell lines [27,31]. Using this approach, we have successfully isolated several novel TAAs such as p62/IMP2 and p90/CIP2A.

An improvement of SEREX approach is to combine it with phage-display technology. The phage-display technology has been widely used since it was first introduced in 1985 [65]. It’s capable to generate and screen peptide libraries to identify ligands for many types of molecules. Screening the phage-displayed libraries needs fewer amounts of sera from patients and has higher efficiency. Tumor cell lines have been used as source of cDNA to construct phage display libraries to identify candidate TAAs in colorectal, breast, prostate, and ovarian cancer [59].

In recent years, proteomics have become a rapidly emerging set of technologies to identify TAAs [66]. The proteome-based methods enable us to screen a large number of sera individually and determine a large number of autoantigens. An advantage of this approach is to distinguish isoforms and detect autoantibodies directly against post-translational modifications (PTMs) of specific targets. In our experience, two-dimensional gel electrophoresis and mass spectrometry can be used to separate lysates from cancer cell lines and identify immunoreactive proteins. In recent several years, we have identified characterized a bunch of protein in HCC and other type of cancer as TAAs with proteomic approach [67,68]. A schematic representation of the approaches for the TAA identification used in our lab has been published [69].

## Using a mini-array of multiple TAAs to enhance autoantibody detection in HCC diagnosis

The unique feature of HCC is that antecedent chronic hepatitis and liver cirrhosis are common precursor conditions and during transition to malignancy some patients develop autoantibodies which are not present during preceding chronic liver disease phase [34,70,71]. It was shown that novel autoantibodies appeared during conversion to malignancy which were not present in the pre-malignant chronic liver disease phase. In HCC, where novel autoantibody responses are detected during conversion to malignancy, it has been proposed that characterization of the autoantigens driving the novel immune responses might provide insights into intracellular proteins participating in mechanisms leading to malignant transformation [1]. Many researchers have been interested in the use of autoantibodies as serological markers for cancer diagnosis, especially because of the general absence or a significantly lower frequency of these autoantibodies in normal individuals and in non-cancer conditions [7,72]. Enthusiasm for this approach has been tempered by low sensitivity when individual antigen-antibody reactions were studied.

Antibody frequency to any individual TAA was variable but rarely exceeded 15-20% [7,73]. We have observed that this drawback can be overcome by using a panel of carefully selected TAAs to achieve the sensitivity and specificity required to make immunodiagnosis a feasible adjunct to tumor diagnosis. This feature is one of the innovative notions in our studies. Previous observations show that antibodies to any individual antigen such as p53 [7], c-myc [7,74], p62 [7] do not reach levels of sensitivity which could become routinely useful in diagnosis. Wang et al developed a phage-display library derived from prostate cancer tissue, and a phage protein microarray, to analyze autoantibodies in serum samples from patients with prostate cancer and controls [72]. In this study, a 22-phage-peptide detector was constructed for prostate cancer screening, with 81.6% sensitivity and 88.2% specificity, which indicated that combinations of multiple antigen-antibody systems might acquire higher sensitivity for diagnosis of cancer [72]. One of our previous studies showed that detection of autoantibodies in cancer can be enhanced by using a mini-array of seven TAAs as target antigens which included c-myc, p53, cyclin B1, p62/IMP2, Koc, IMP1 and survivin [7,73]. Antibody frequency to any individual TAA ranged from 10.8 to 24.6% in HCC. With the addition of TAAs to a final total of seven antigens, there was a stepwise increase of positive antibody reactions up to 56.9% in HCC. In our further study, p16, a cyclin-dependent kinase inhibitor, was evaluated as a TAA and added into our previously constituted mini-array of seven TAAs. Antibody frequency to any individual TAA in HCC was variable from 9.9%-21.8% but rarely exceeded 20%. There was a stepwise increase of positive antibody reactions up to 59.9% with successive addition of TAAs to a final of eight antigens, which was significantly higher than the frequency of antibodies in chronic hepatitis (20%), liver cirrhosis (30%) and normal individuals (12.2%). The sensitivity on diagnosing HCC was 59.9%, and the specificity was 87.8%, 80% and 70%, respectively, compared to normal individuals, chronic hepatitis and liver cirrhosis [75]. The data from our studies indicates that the combination of antibodies might acquire higher sensitivity for diagnosis of cancer.

Nevertheless, in the selection of different antigen-antibody systems, some of the antigens may turn out to be more specific for a certain type of cancer while others may be not. It is conceivable that autoantibody profiles involving different panels or arrays of TAAs might be developed and the results could be useful for diagnosis of certain types of cancer. We stress the notion that panels of “customized” TAAs should be used for different types of tumor and that these customized panels should be rigorously tested for sensitivity and specificity not only against other tumors but also against other diseases conditions. In the case of HCC, the natural conditions could be chronic hepatitis and liver cirrhosis. The basis for the notion of the necessity of customized panels of TAAs is based not only on empirical observations but also on retrospective analysis of our own data learned from previously published work [7,34,71,73]. The main focus of our studies is to identify a specific panel of TAAs for HCC and compare and contrast this with antigen panels associated with chronic hepatitis and liver cirrhosis.

## Possibility for anti-TAAs antibody as prognosis indicator

It is of great importance to find early detection of cancer in clinical oncology, which allows an effective and probably curative therapeutic management [10]. In the meanwhile, the prognosis of a patient diagnosed with cancer is considered as the chance that the disease will be cured and the patient will recover. It is known that various factors can affect the prognosis of a patient with cancer, including the biological and genetic properties of the cancer cells, which are also known as biomarkers and can be determined by certain tests. The potential utility of anti-TAAs antibody as early cancer biomarker or indicators of disease prognosis has been explored, since these anti-TAA antibodies are generally absent, or present in low frequency in normal individuals and in non-cancer conditions.

More and more evidences have shown that the immune system of the cancer patient is able to sense abnormalities in structure, function, intracellular location and other alterations of cellular participants in tumorigenesis [2]. Although most current studies are focusing on cancer autoantibodies as diagnostic biomarkers, it is likely that autoantibodies can also be used as monitors of therapeutic response, thus contributing to cancer prognosis. It is hypothesized that in a patient where a certain anti-TAA antibody is detected, change in antibody levels might reflect change in tumor status or tumor burden related to therapy [2].

Currently, although the continuous development and progress have been made in early diagnosis and treatment of cancer, however we are still facing problems to find sensitive and accurate biomarkers for cancer diagnosis, prognosis and treatment. Molecular studies using TAA-based arrays have identified several cancer related proteins involved in cell proliferation and metastasis during the process of tumorigenesis [76]. Such proteins have been detected in patients’ serum, which distinguish diseased individuals from healthy controls. Using TAA-array technique might be a useful tool to determine the groups of cancer patients with certain clinical outcomes, and consequently benefit to personalized treatment. Thus, it is of great importance to determine reliable biomarkers with high prognostic value.

## Conclusions

In the past few years, the potential utility of autoantibody to TAAs as cancer biomarker for early detection or as indicators of disease prognosis has been explored. Our efforts will be aimed at increasing both the sensitivity and specificity of antibodies as markers in HCC by expending TAA array to include antigens which might be more selectively associated with HCC and not with others. It is expected that our mini-array of multiple TAAs can be used as a novel non-invasive approach to identify HCC in normal population and also in high-risk individuals such as patients with chronic hepatitis and liver cirrhosis. We predict that, in the next five years, the research on the cancer TAA autoantibody biomarkers will become a challenging field. With the data at hand, we know that many TAAs are not unique to any particular type of solid tumor and this should not be unexpected since tumorigenesis pathways are common to many tumors. Waiting for the generation of TAA arrays for immunodiagnosis of specific cancers, however, should not preclude taking advantage of the sensitivity of the anti-TAA/TAA systems in the initial screening process for early detection of cancer. Important additional studies will be needed to determine whether antibodies to a mini-arrays of multiple TAAs might be useful in identifying in early stage of certain types of other cancer in high-risk individuals.

## Abbreviations

AFP: Alpha fetoprotein; CAPER: Co-activator of activating protein-1 and estrogen receptors; CIP2A: Cancerous inhibitor of PP2A; CRD-BP: Coding region determinant-binding protein; HCC: Hepatocellular carcinoma; IIF: Indirect immunofluorescence; KOC: K-homology overexpressed in cancer; SEREX: Serological analysis of recombination cDNA expression libraries; TAAs: Tumor-associated antigens; ZBP1: Zip-code binding protein 1.

## Competing interests

The authors declare that they have no competing interests.

## Authors’ contributions

LD, NL and ML have been involved in drafting the manuscript. JZ has revised the manuscript critically and given final approval of the version to be published.
